# Localization of Free-Field Sound Sources in the Chronic Phase of Mild Ischemic Stroke

**DOI:** 10.1177/23312165261446384

**Published:** 2026-05-14

**Authors:** Mathias Dietz, Anna Dietze, Peter Sörös, Karsten Witt, Henri Pöntynen

**Affiliations:** 1 Department of Medical Physics and Acoustics, 11233 University of Oldenburg, Oldenburg, Germany; 2 Cluster of Excellence “Hearing4all”, University of Oldenburg, Oldenburg, Germany; 3 Research Center Neurosensory Science, University of Oldenburg, Oldenburg, Germany; 4 Department of Neurology, School of Medicine and Health Sciences, University of Oldenburg, Oldenburg, Germany

**Keywords:** sound localization, binaural hearing, psychoacoustics, brain lesions, magnetic resonance imaging, stroke

## Abstract

Accurate sound localization relies on the transformation of binaural cues into stable spatial representations, yet the neural mechanisms supporting this process remain incompletely understood. Mild stroke provides a unique opportunity to study the vulnerability of auditory spatial processing within distributed neural networks. We investigated azimuthal sound localization in chronic-phase survivors of mild stroke without hearing aid use using broadband, low-frequency, and high-frequency noise and compared it against a headphone-based lateralization task. Most patients exhibited localization accuracy comparable to healthy listeners. However, 4 of 14 patients showed a striking alteration of auditory spatial perception: instead of a continuous mapping of azimuth, responses clustered into bi- or trimodal (left-center-right) categorical patterns. Comparable deficits were also observed in one of the five age-matched control participants. To our knowledge, such localization patterns have not been reported in listeners without neurological disease. Atypical localization was most pronounced with low-frequency stimuli, suggesting a different role of the respective binaural cues in forming a spatial representation of sound. Differences between loudspeaker-based localization and existing headphone-based lateralization data further suggest that these paradigms engage distinct auditory spatial representations. The findings support the view that auditory space is constructed through higher-order, supramodal spatial mechanisms that are particularly vulnerable to right-hemispheric damage. Overall, the data highlight the challenges of quantifying spatial hearing of stroke survivors both on an individual and on a population level. Challenges include previously undocumented stimulus and method dependencies, an unknown mixing of auditory and neurological factors, and the absence of normative data for a nonstroke control cohort.

## Introduction

Spatial hearing along the azimuthal dimension relies on accurate encoding of sound level and temporal information in two ears, on binaurally sensitive neurons in the brainstem encoding the respective interaural level difference (ILD) and interaural time difference (ITD), and on the integrity of neural computations in more central structures. While many studies have investigated the effects of peripheral deficits on auditory perception, the effects of damage to central structures remain less explored and poorly understood. At the population level, stroke represents a prevalent pathology whose individual and often multimodal effects are commonly task-dependent and hemisphere specific. Visual neglect of the contralesional hemifield, for instance, is well-studied (e.g., Ting et al., 2011), but also auditory hemifield neglect has been observed in several studies (e.g., Dietz et al., 2014; Gokhale et al., 2013; Gutschalk & Dykstra, 2015).

Previous studies have revealed localization deficits after brain lesions (for instance, Litovsky et al., 2002; Sanchez-Longo & Forster, 1958; Tanaka et al., 1995; Zatorre & Penhune, 2001). Unsurprisingly, the severity and type of spatial or binaural hearing deficits varies widely across subjects depending on the size and location of the lesion (e.g., Tanaka et al., 1995). Specifically, localization deficits have been reported to be more frequent and severe in patients with right-hemispheric lesions (Dietz et al., 2014; Haeske-Dewick et al., 1996; Sonoda et al., 2001).

The small body of literature on localization deficits after stroke is complemented by studies that focus on the effect of stroke on ILD and ITD processing. Those studies typically employ headphone stimulation where the binaural cues are manipulated independently of one another (Aharonson et al., 1998; Dietze et al. 2022, 2024; Furst et al., 2000; Spierer et al., 2009). Such stimuli result in spatial percepts that are perceived as intracranial (inside the head) rather than outside the surrounding environment. Therefore, the term “lateralization” is used to refer to the left-to-right position of intracranial spatial percepts associated with headphone stimulation, and the term “localization” refers to spatial perception of sounds that are perceived to be outside of the head.

Aharonson et al. (1998) investigated lateralization perception in patients with pontine lesions and reported two general trends of abnormal binaural processing. Seven of 13 stroke patients perceived all stimuli (regardless of ITD or ILD) at or near the center of the head, suggesting a reduction or loss of sensitivity to binaural cues. Three patients perceived all stimuli toward one or both sides of the head. Only three of the 13 tested stroke patients did not show signs of abnormal lateralization. In Furst et al. (2000), the effects of brainstem lesions on binaural processing were assessed using a lateralization task and measurements of discrimination thresholds for interaural disparities. These experiments showed that spatial processing was impaired when the lesion affected structures along the auditory pathway. Further, qualitatively different impairments were observed depending on the affected structures. For instance, patients who had suffered a lesion to structures rostral to the superior olivary complex displayed a lateral bias in the lateralization task whereas patients with caudal pons lesions displayed a compression of auditory space, so that most stimuli were perceived to be near the auditory midline. Cortical level lesions have also been shown to affect spatial perception. In line with the localization studies mentioned above, Spierer et al. (2009) showed that both ITD- and ILD-driven lateralization percepts were more affected by cortical impairments in the right hemisphere. Recently, Dietze et al. (2022) investigated lateralization perception in a large cohort (N = 50) of mild stroke survivors in the acute phase of the pathology. The affected neural structures included a wide range of cortical and subcortical lesion sites. As in Furst et al. (2000), lateralization perception was found to be impaired in most patients with lesions along the auditory pathway. Similarly, different lesion sites were associated with qualitatively different patterns of impaired lateralization. For instance, brainstem-level lesions led to a bimodal (left-right) or trimodal (left-center-right) lateralization response and lesions affecting thalamic structures were associated with a lateralization bias. Lateralization of contralateral stimuli was impaired in some instances. In line with Spierer et al. (2009) lesions in the right hemisphere had a stronger effect on lateralization than left-hemispheric lesions. On the brainstem lesion level, however, the four patients with a right brainstem lesion are, on average, less impaired than the three patients with a lesion in the left brainstem.

Theoretically, there should be a direct relation between lateralization and localization impairment, as both are exclusively or predominantly facilitated by the same interaural cues. This is both textbook understanding (Litovsky et al., 2021) and the underlying assumption of sound localization models (Dietz et al., 2011; Faller & Merimaa, 2004). Lateralization and localization tasks differ in terms of the acoustic stimulation as well as in more abstract methodological aspects. It remains unclear to what extent these differences may be reflected in perceptual data obtained from the two tasks.

While lateralization experiments offer precise control over the stimulus parameters, they suffer from a lack of ecological validity. Sounds encountered in daily life provide a combination of spatially congruent directional cues that were absent in the above-mentioned headphone stimulations. It is therefore not clear to what extent performance in headphone-based lateralization experiments transfers to localization tasks, where stimuli are a step closer to the sounds experienced outside of laboratory conditions. Similarly, it is unclear to what extent spatial hearing deficits affecting only a single cue modality could remain undetected in loudspeaker experiments, where multiple spatial cues are available. It has been suggested that headphone experiments may be more sensitive to detecting spatial processing impairments that may remain undetected in free field localization tasks (Spierer et al., 2009; Tanaka et al., 1999). Thus, one aim of the present study was to assess sound localization abilities in the same cohort of mild stroke survivors that was also tested for ITD- and ILD-based lateralization. The second aim was testing the qualitative congruence of localization and lateralization.

## Methods

### Participants

Fourteen stroke patients (mean age of 58.7 years, SD: 16.2 years, 3 female, 11 male) participated in the localization experiment after providing written informed consent. Four of the stroke patients were under the age of 50 (47, 43, 30, and 28 years); the mean age of the remaining 10 older stroke patients was 67.4 years (SD: 7.6 years). Five older control subjects (66.2 years, SD: 8.2 years, 4 female) were age-matched to the latter group. Lastly, we included six young normal-hearing subjects (mean age of 24.2 years, SD: 2.9 years, 3 female), as a secondary reference group. For the remainder of the text, participants who had suffered a stroke will be referred to as patients, and the remaining participants as young reference and (age-matched) control subjects, according to their respective age cohort. The study was approved by the Medical Research Ethics Board of the University of Oldenburg, Germany. The patients participating in this study were a subset of the patients initially recruited from the stroke unit of the Evangelisches Krankenhaus, Oldenburg, Germany for the study described in Dietze et al. (2022), and the longitudinal study described by Dietze et al. (2024). Recordings were obtained directly after the “chronic-phase” measurements of Dietze et al. (2024), for the subset of participants that volunteered to also do the localization task. The age-matched control subjects were either partners of the stroke patients who accompanied them to the testing laboratories or externally recruited. The young reference group consisted of university students from the University of Oldenburg, many of whom had previous experience with psychoacoustic experiments. The inclusion criteria for the control groups were no wearing of hearing assistive devices, no severe hearing loss or deafness, and no self-reported history of stroke or other neurological diseases.

### General Assessment, Magnetic Resonance Imaging, and Audiometry

A brief overview of the pre-experimental screening of the stroke patients is provided below. For a detailed account of these procedures, see Dietze et al. (2022). The severity of the stroke was assessed in the acute phase using the National Institute of Health stroke score (National Institute of Neurological Disorders and Stroke [NIHSS], 2019) and the location and volume of the stroke lesions were characterized using the 1.5-T magnetic resonance imaging (MRI) data obtained during the clinical routine for all participants recruited from the hospital stroke unit. Patients were screened for mild cognitive impairment or dementia using the Montreal Cognitive Assessment (MoCA, Nasreddine et al., 2005). In addition, the patients completed a multiple-choice vocabulary intelligence test (the German MWT-B, Lehrl, 2005) and were screened for depression using the short version of Beck's Depression Inventory (BDI, Beck et al., 2013). As described in Dietze et al. (2024), the MoCA, MWT-B, and BDI assessments were collected longitudinally on each of the three measurement appointments corresponding to the acute, subacute, and chronic phases of stroke. These tests were not performed on the control cohort. For all study participants audiometric thresholds were measured at the following kHz-frequencies: .125, .25, .5, .75, 1.0, 1.5, 2.0, 3.0, 4.0, 6.0, and 8.0.

### Stimuli

**Figure 1.**
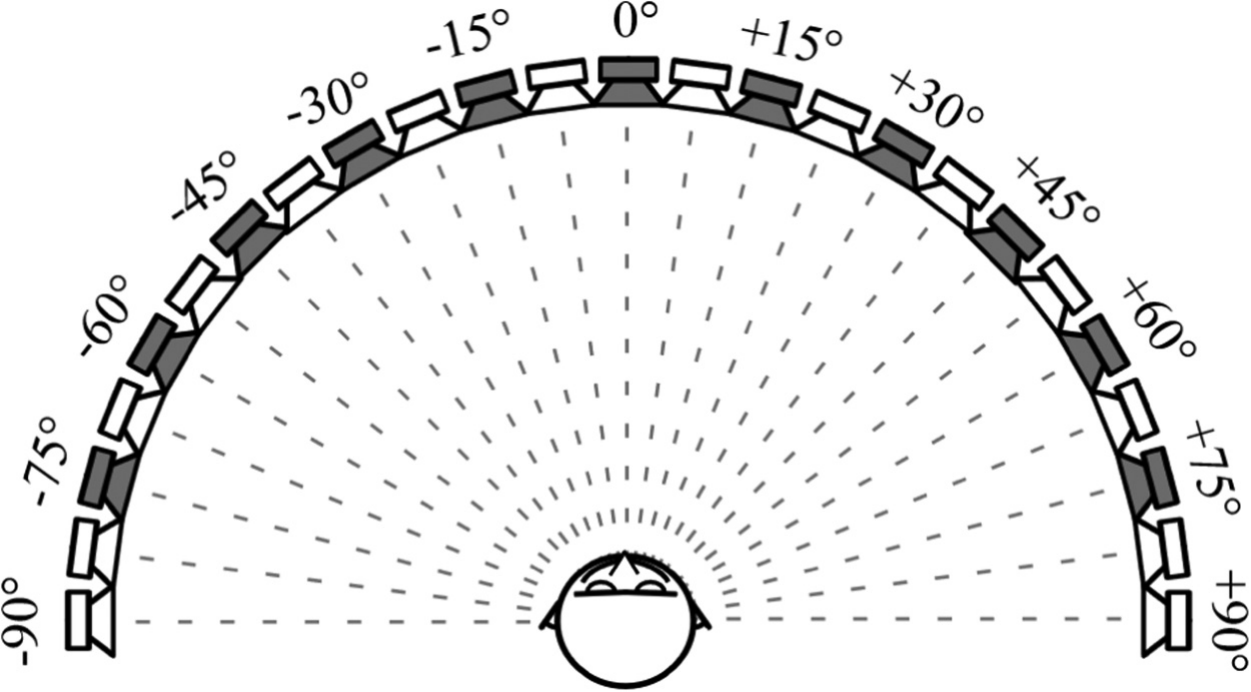
Experimental setup of the localization experiment. Subjects were seated at the center of a circular arc of 25 loudspeakers spanning the frontal hemifield. The angular separation between adjacent loudspeakers was 7.5 deg. In each trial, a stimulus was presented from one of the loudspeakers marked in gray. The subjects reported their localization percepts verbally by indicating the label on the loudspeaker that they thought had produced the stimulus.

Three different stimulus types derived from pink noise were used: low-pass noise (.125–.5 kHz, referred to as “LP” condition), high-pass noise (2.0–8.0 kHz, HP condition), and broadband noise (.125–8.0 kHz, BB condition). All stimuli had a total duration of 200 ms and were gated with 20ms raised-cosine onset and offset ramps. All stimuli were presented with a sample rate of 44.1 kHz. Stimulation level for each of the three spectra was 70 dBA as measured with a sound level meter at the listening position at the center of the loudspeaker array. The three stimulus types were presented six times each from 11 different azimuth angles (see the gray loudspeakers in Figure 1) in fully randomized order.

### Experimental Apparatus and Procedure

The localization experiment was conducted in an anechoic chamber fitted with a multichannel loudspeaker system. The subjects were seated at the middle point of a circular arc (radius: 2.5 m) of 25 loudspeakers spanning ±90 deg. of the frontal azimuthal plane (see Figure 1). The angular separation between adjacent loudspeakers was 7.5 deg. The front faces of the loudspeakers were labeled with ascending numbers from 1 to 25 so that the leftmost loudspeaker had the label 1 and the right-most loudspeaker had the label 25. In each trial, a stimulus was presented from one of the 11 source positions (see the gray loudspeakers in Figure 1), at 0, ±15, ±30, ±45, ±60, or ±75 degrees azimuth. Participants were not informed that sounds were only presented from 11 of the 25 speakers. Prior to each trial, an optical camera system (“Miqus” cameras from Qualisys) was used to monitor the orientation of the participant's head to ensure that they were facing the loudspeaker at the middle of the arc. The head orientation was scanned at a rate of 100 Hz. A computer screen providing real-time feedback of the participant's head orientation was attached below the loudspeaker at 0 deg. azimuth to facilitate rapid, intuitive adjustment of the head position. A trial could be initiated if the participant was facing the middle loudspeaker within bounds of ±3 deg. in azimuth and elevation. Similarly, the tracker was used to limit the side-to-side as well as front-to-back displacement of the subjects within ±5 cm of the center position. Once the subject was facing the middle loudspeaker, a trial could be initiated by the experimenter accompanying the participant in the test chamber. The headtracking data was streamed into the Matlab script that controlled the experiment over a local area network connection using the “Qualisys Track Manager (QTM) Connect for Matlab”—extension for Qualisys Track Manager software that processed the orientation data from the camera system. The camera system was only used to ensure that the subject was oriented appropriately before a trial was initiated.

After each trial, the task of the participants was to verbally report the label (number) of the loudspeaker that they thought had produced the stimulus. Participants were instructed not to move their head during the presentation of the stimuli but were free to move their head after stimulus offset. This was done to encourage the participants to check the labels of the lateral loudspeakers that were left outside of the field of view when facing the middle loudspeaker. Once the participant's response had been registered by the experimenter, they re-oriented toward the loudspeaker at 0 deg. azimuth to initiate a new trial. The experiment progressed in this manner until all 198 trials (6 repetitions, 3 spectra, 11 azimuths) had been presented. All trials were presented within a single fully randomized experimental block. The experiment was implemented in Matlab using the psychophysical measurement toolbox “AFC” (Ewert, 2013). Before starting the experiment, all participants completed a small number of practice trials (typically less than 10) to familiarize themselves with the experimental procedure. The practice trials were not included in the results of the participants.

### Localization and Audiogram Metrics

Localization performance was quantified by the root mean square error (RMSE), signed bias and standard deviation (Hartmann, 1983). All three metrics were derived for each patient and each of the three stimuli as the mean across the 11 tested target azimuths. While RMSE is a metric that represents the overall localization accuracy the signed bias gives an estimate of a constant error that is sensitive to direction. The standard deviation of the six responses to each azimuth is a measure of response variability.

The localization performance metrics were then related to two metrics extracted from the audiograms, namely: mean hearing level (binaural pure tone average, PTA) and hearing loss asymmetry (measured as audiogram asymmetry, delta PTA). Delta PTA was computed as the difference between the left and right pure tone audiograms so that a positive delta PTA corresponds to more hearing loss in the left ear. For the mean hearing level metric (PTA), a single value characterizing the subject's overall hearing loss was obtained by averaging over the audiometric thresholds of both ears. For both PTA and delta PTA, averaging was performed across all audiometric frequencies within the range of the respective stimulus, against which the comparison is made. For the low-pass condition, audiogram values at 125, 250, and 500 Hz were used, for the high-pass condition the values were from 2, 3, 4, 6, and 8 kHz, and for the broadband condition, all measured frequencies of the audiogram were used (0.125–8 kHz). Subsequent correlation analysis was performed only for the stroke patients. As we cannot expect a linear relation we performed a rank correlation and because of the small sample size we calculated Kendall's τ. For our N = 14 statistical significance at the 0.05 level is reached for τ > 0.4, so that only the τ-values are stated.

## Results

### MRI Data

**Figure 2.**
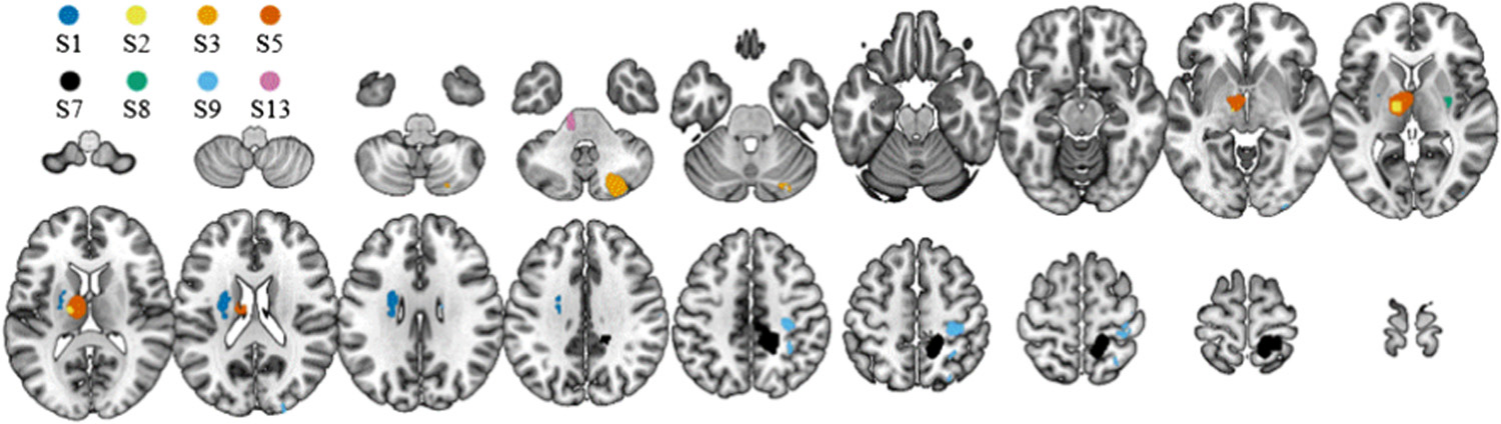
Exemplary lesion maps for a subset of the stroke patients. S1 (dark blue): left basal ganglia. S2 (yellow): left thalamus. S3 (orange): right cerebellum. S5 (red): left thalamus. S7 (black): right hemisphere, multiple lesions. S8 (green): right basal ganglia. S9 (light blue): right hemisphere, multiple lesions. S13 (pink): left brainstem.

**Figure 3.**
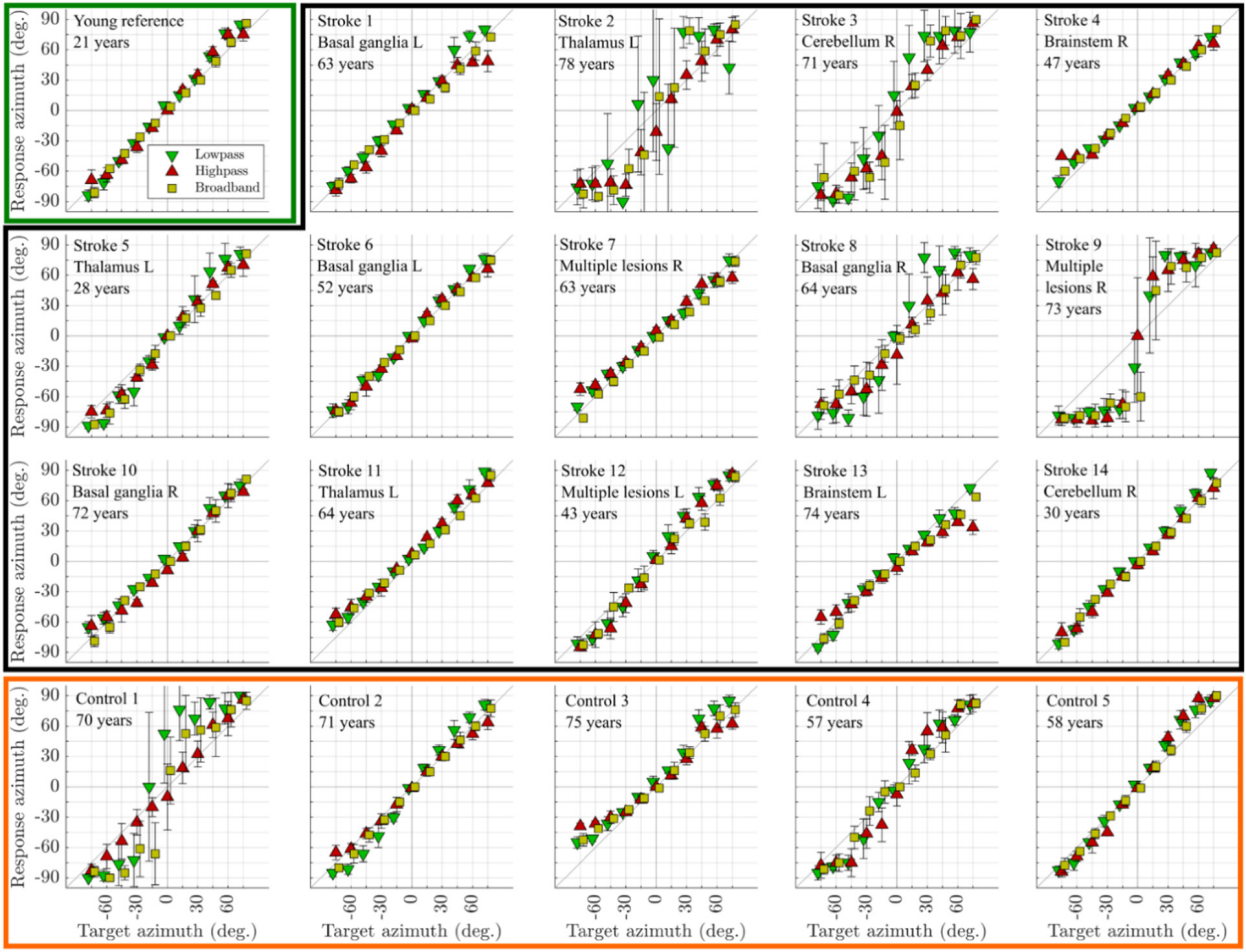
Individual localization results for the stroke cohort and selected control subjects. The first panel shows the results from an exemplary young reference subject (delineated by green borders). The panels delineated by the black border represent the results of the stroke patients. The bottom row shows the results from the age-matched control group (delineated by orange borders). Error bars represent the standard deviation across six trials.

Figure 2 shows the locations and relative sizes of the stroke lesions for a subset of the stroke patients. The lesion locations for all patients are shown as text insets in the corresponding localization response plots in Figure 3.

### Localization

The localization results are shown in Figure 3. In addition to the results from the 14 stroke patients, Figure 3 shows the corresponding data from one representative subject in the young reference cohort (panel 1) and the five control subjects in the age-matched nonstroke cohort (bottom row).

**Figure 4.**
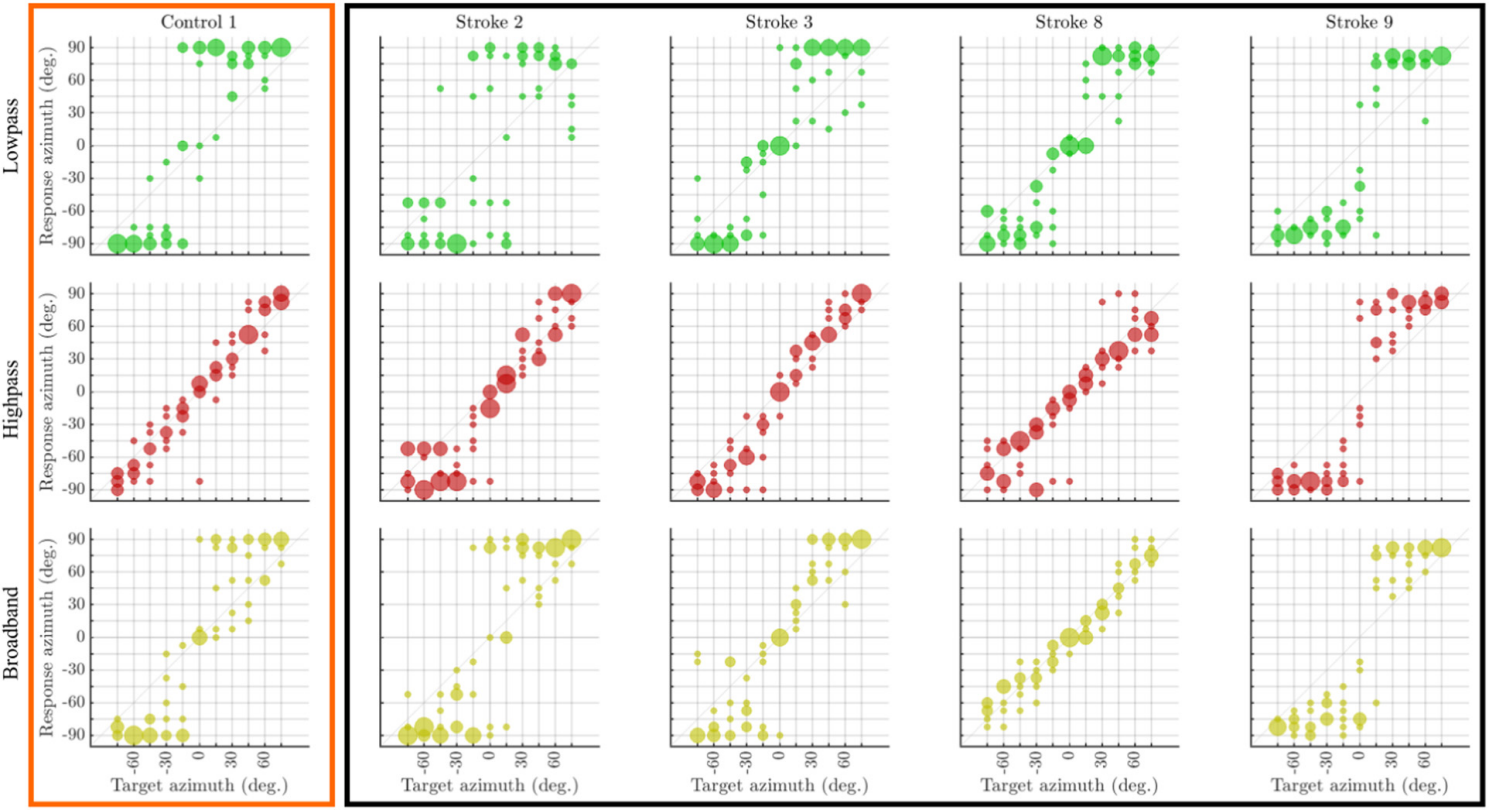
Bubble plots of the responses to the three stimulus conditions for the five subjects whose localization performance was aberrant. The first, second, and third rows show the data for the low-pass, high-pass, and broadband conditions, respectively. Orange and black borders delineate the results from participants in the control and stroke patient groups, respectively.

The majority of patients show good localization performance, and at a general level, their results are qualitatively similar to those obtained from the control groups. However, the performance of patients 2, 3, 8, and 9 as well as control 1 stands out as aberrant. For instance, these patients gave inconsistent responses across the six repetitions of the stimuli at different azimuths, especially for stimuli presented from frontal speakers. Here, this tendency manifests as large error bars. Since the localization patterns of these five subjects are poorly captured by the visualizations based on the mean and standard deviation used for the other subjects, Figure 4 shows the corresponding bubble plots for these subjects. The bubble plots show that these subjects tend to localize all stimuli either at the far left or the far right, especially the low-frequency noise but also the broadband noise. Patient 9 is an extreme example of this localization behavior, as he mostly gave responses beyond **±**60° for all three noise types and even for frontal presentation. For patients 2, 3, and 8 and for control 1, sometimes a third response area at frontal positions is visible and high-pass noise is localized more continuously with fewer errors.

#### Localization Deficits Occur Only in the Elderly Listeners

**Figure 5.**
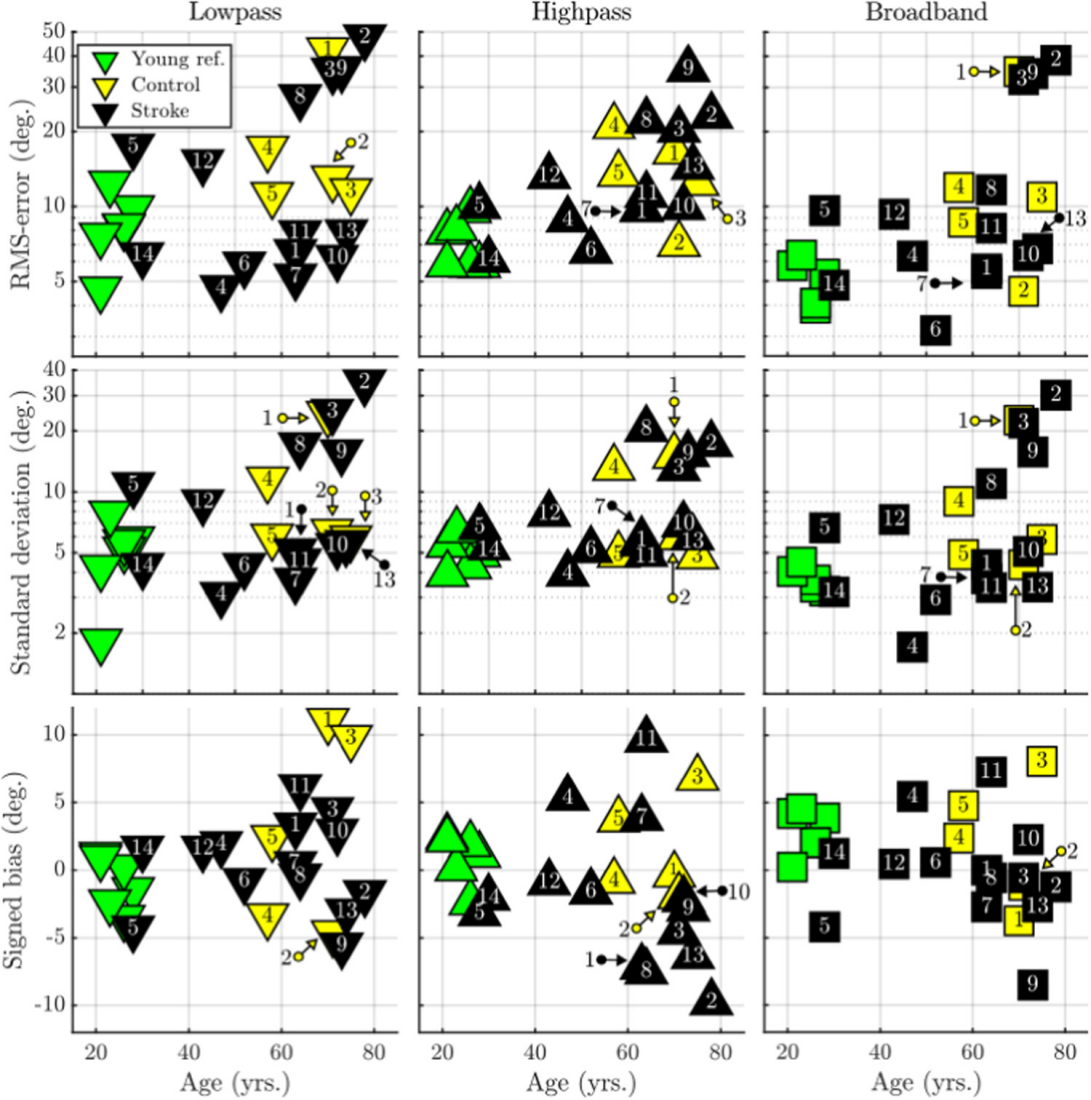
Scatterplots of age versus localization metrics. The subject number labels for overlapping data points have been indicated with color-coded arrows; yellow arrows refer to participants in the control group and black arrows to stroke patients. The six young reference listeners are marked with green symbols, but with numbers, due to their large overlap.

Figure 5 shows scatterplots of the age of the subjects versus the localization metrics obtained in the three stimulus conditions. The stroke cohort does not stand out from the control cohort in any of the nine panels, suggesting that performance did not significantly differ from the control cohort. However, the group of five subjects (stroke patients 2, 3, 8, 9, and control subject 1), whose localization performance was clearly different from the rest of the subjects in Figure 3, tend to cluster together in the upper right corner of some of these plots. This indicates that while the localization performance of these subjects was poor, they were also among the oldest participants in the study. For the low-pass and broadband condition the rank correlation between the RMSE and age was only an insignificant trend (τ = 0.37 and 0.39, respectively), but for the high-pass noise condition the correlation was significant (τ = 0.52). The MoCA scores of patients 2, 3, 8, and 9 were 26, 25, 26, and 27, respectively, which were only marginally below the average MoCA score of 27 from the 10 other patients. The localization performance of many of the participants in both the stroke, and nonstroke cohort are comparable to those of the participants in the young normal-hearing group.

#### Hearing Loss Asymmetry Does Not Consistently Relate to Signed Bias

**Figure 6.**
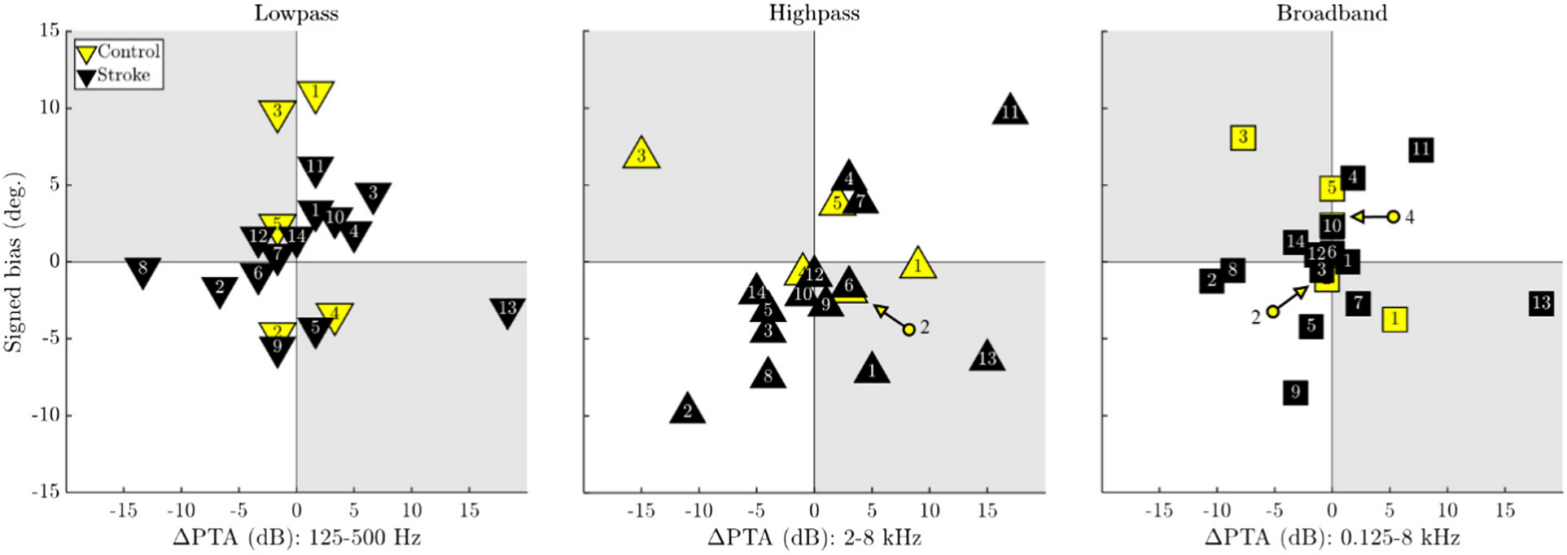
Scatterplots of hearing loss asymmetry (delta PTA) versus signed bias. The subject number labels for overlapping data points have been indicated with color-coded arrows. The gray quadrants denote areas where signed bias is in direction opposing the participant's hearing loss asymmetry.

Asymmetric hearing loss is known to cause a sound localization bias in some individuals (Florentine, 1976; Zimmer et al., 2024). Therefore, prior to claiming a potential localization bias or error to be stroke-related, we analyzed the influence of hearing asymmetry for the two older cohorts in the scatterplots of Figure 6. Eighty-eight percent of band-averaged audiometric asymmetries were below 10 dB. Exceptions are patient 13, who had 15 to 20 dB asymmetry throughout the tested frequencies, patient 8 who had a 10 to 15 dB asymmetry at low frequencies, and patients 8 and 11 as well as control subject 3 who had 10 to 18 dB asymmetries at high frequencies. Of these five individuals with asymmetric hearing thresholds, patients 2 and 11 had a localization bias toward the ear with lower audiometric thresholds, that is, audiometric asymmetry alone is a plausible explanation for the bias. Patient 8 had a very small bias in this direction as well. However, control subject 3 and patient 13 had a bias that is inconsistent with their hearing asymmetry. They localized some sounds more toward the side of their poorer hearing ear. Last, patient 9 has a consistent bias of up to 8° toward the left, without a nominal delta PTA. However, this 73-year-old patient has a considerable 65 dB HL hearing threshold in both ears at 4 kHz, and bias-consistent asymmetries at and around 1 kHz. For all three noise types there was a positive rank correlation between ΔPTA and signed bias. While it never reached significance, there was a clear trend in the high-pass condition (τ = 0.37), qualitatively in line with Florentine (1976). As such, audiometric asymmetries have to be considered before any small biases can be confidently related to a stroke lesion location.

#### Localization Deficiencies Exist Even in Absence of Hearing Loss or Stroke

**Figure 7.**
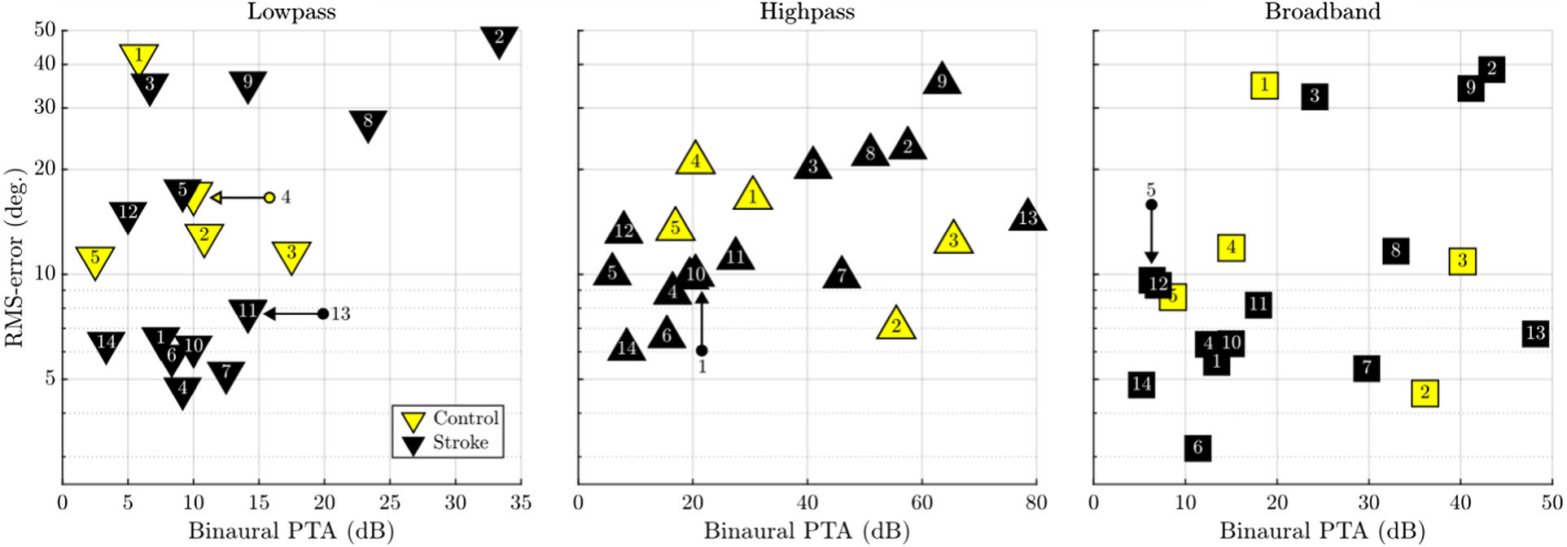
Scatterplots of binaural PTA versus RMSE. The subject number labels for overlapping data points have been indicated with color-coded arrows. PTA=pure tone average; RMSE= root mean square error.

As in the previous two subsections, before relating any elevated RMSE to the patient's stroke lesion, the confounding factor of hearing loss has to be considered. While the previous analysis was concerned with asymmetries and directional bias, this final check relates the ear-averaged pure-tone average threshold (binaural PTA) to the individual RMSE (Figure 7). The only significant correlation between RMSE and hearing loss was found in the high-pass condition (τ = 0.56), where, expectedly, the largest values of hearing loss were observed. While the broadband-based correlation fell short of statistical significance, we note that the four patients with the most atypical localization (patients 2, 3, 8, and 9) all have worse than median audiometric thresholds, but that is not the case for control subject 1. In the low-frequency comparison, where the atypical localization was most pronounced, patient 3 and control subject 1 have very good and better than average audiometric thresholds. On the other hand, two other atypical localizers, patients 2 and 8, happen to be the only two patients with low-frequency hearing loss of more than 20 dB HL.

#### Localization is Not Always Consistent With Lateralization Performance

**Figure 8.**
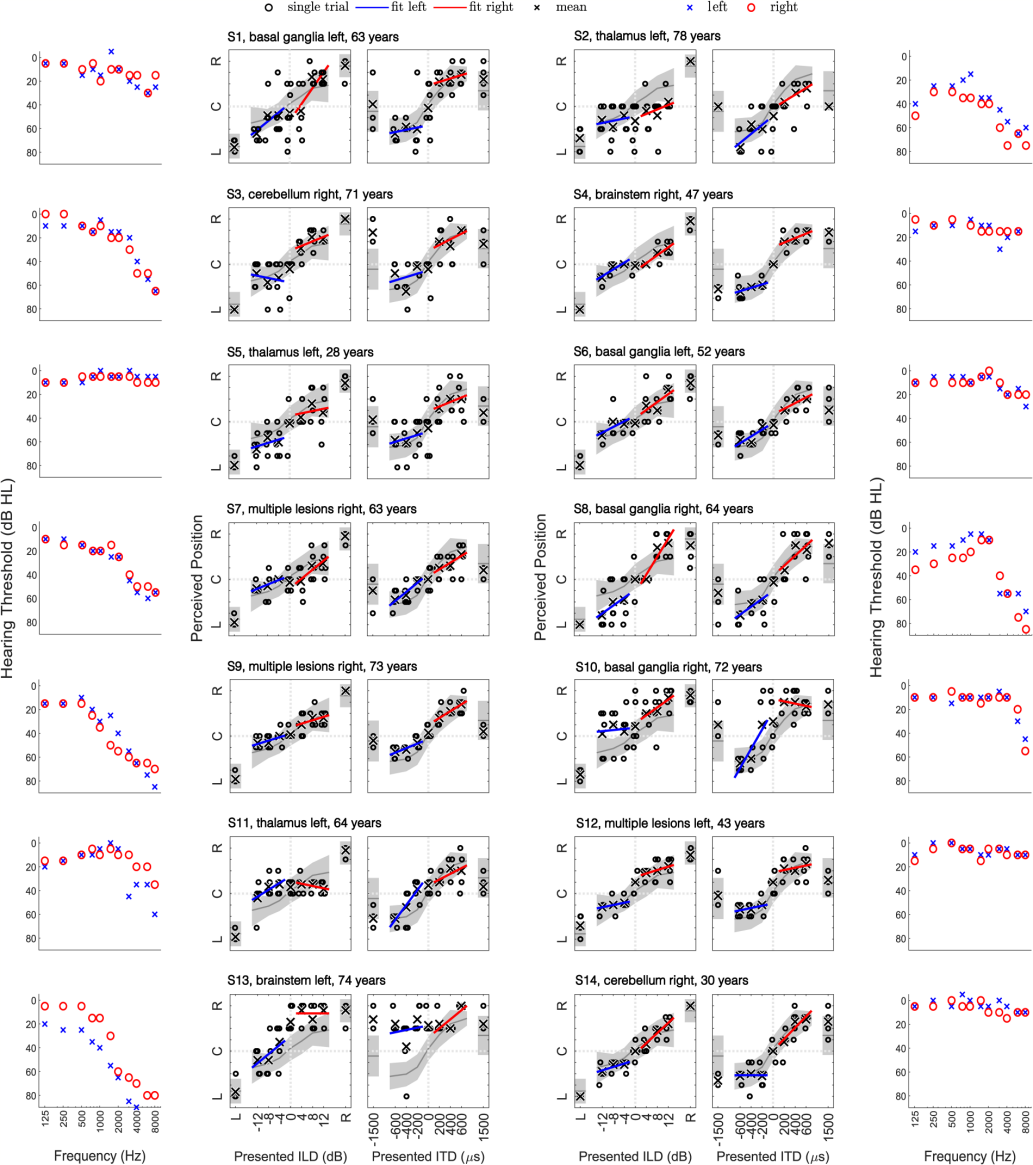
Lateralization data and audiograms of the 14 stroke patients who participated in the localization experiment. Note that the subject identifiers are not identical across the two studies reported here and in Dietze et al. (2024). The shaded area indicates the range of control subject performance.

The current localization data was gathered on the day of the third measurement (chronic-phase) of the patients in Dietze et al. (2024). As such, both localization and lateralization data gathered within a few hours are available for the stroke patients included in this study. These data, along with the audiograms, are shown in Figure 8. Focus is on the comparison between localization of low-frequency noise and ITD-based lateralization, as these conditions can be expected to provide the most similar cues—namely low-frequency ITDs. Note that the stimulation conditions between the two experiments were similar but not identical. 2-oct. pink noise between 125 and 500 Hz was used in the low-pass condition of the localization experiment while 1-oct white noise between 333 and 666 Hz was used in the headphone experiment. We first focus on the four patients that has abnormal localization.

Both patients with a trimodal localization response (S3 and S8), also have a somewhat trimodal response in case of ITD-based lateralization. Patient S2 (top right of Figure 8) only responded with the odd numbers 1, 3, 5, and 7 in case of ITD-based lateralization, never with an even number or the right-most number 9. Responses change systematically but with substantial variability from left to center-right with increasing ITD. For right-only presentation this patient responds with 9. Notably, the low-frequency localization data also reveals a quadru-modal pattern but instead of a left bias now with two clusters on either side and almost no central responses. Patient 9, who has the most pronounced bimodal localization performance for all three stimulus types, performed much better in both lateralization tasks, with no indication for a bimodal response at all.

Of the 10 patients that performed normally in localization, seven also performed quite normally in the lateralization task. Only patients S10 and S13 have a strong right-bias in both ITD- and ILD-based lateralization but not in localization. The lateralization bias of S13 could be explained based on the asymmetric hearing loss but it is absent in the localization data. S5 had a very noisy response in the lateralization task, and a rather normal localization response, albeit also a little noisier than the average.

In an attempt to summarize this comparison into categories, we leave it to the reader to judge if S2 and S5 are consistent or inconsistent, as we see arguments either way. Of the other 12 patients, seven had accurate localization and normal ITD-based lateralization and two had similar trimodal response patterns in both tasks. Three patients were clearly inconsistent: two had a lateralization bias but unbiased localization and one had normal lateralization but a very bimodal localization response.

## Discussion

For healthy listeners of all ages sound localization is typically very accurate. For example, Dorman et al. (2016) reported from 12 listeners between 51 and 70 years to all have RMSE well below 10° for broadband noise. Freigang et al. (2014) reported from 64 listeners between 65 and 83 years to all have RMSEs below 12° for near-frontal sound sources of low-frequency noise and all but one listener had errors below 14° for low-frequency noise presented from 30° to the side. These small RMSEs require virtually all responses to be close to the true source, that is, lined up along the main diagonal when visualized as in Figures 3 and 4. Such typical responses were observed in all our young listeners, in four of our five control listeners and in 10 of 14 stroke patients. In contrast, some stroke survivors, especially those with right hemispheric lesions, have been reported to perceive perifrontal and sometimes frontal sounds as originating from lateral positions (Haeske-Dewick et al., 1996; Sonoda et al., 2001). We also found such bimodal (left or right) or trimodal (left-center-right) response patterns in four of the 14 stroke patients and, unexpectedly, in 1 older control listener. These response patterns result in overall RMSEs beyond 20° and near-frontal sources had RMSEs of up to 69°.

The older control subject with a trimodal response and an RMSE exceeding 30° with broadband and low-pass noise, is therefore very atypical, with errors several times larger than the worst of 76 elderly listeners from Dorman et al. (2016) and Freigang et al. (2014) combined. When 76 of 76 listeners are good localizers that means that we can expect at least 96% of a comparable population to be good localizers (Brown et al., 2001). Therefore, we kept our control group so small. We did not expect to learn anything important and only required them to confirm that literature-like performance is obtained with our setup and our choice of stimuli. Having only four of the five good listeners in our sample is not in contradiction to a 96% prevalence, of course, but the atypical data from control subject 1 raises the question about its cause. In search for explanations, we learned that mild strokes sometimes go unnoticed, with estimates ranging up to one in three people aged 70 or older (Lim & Kwon, 2010). The question is then, why have studies with nonstroke elderly listeners not reported similar bimodal or trimodal response patterns or at least RMSEs that are large enough to be compatible with such patterns. In the absence of MRI scans and other medical and cognitive testing of our control group, our best guess is to assume that control 1 has an unknown pathology that compromised the localization performance. In other words, for the remainder of this discussion, we assume that listeners with age-typical hearing and no brain lesions or neurological disorders can localize sounds with an average RMSE of 5° to 10°, but no more than 20° for low-pass noise or 12° for broadband stimuli. This assumption is best in line with both the literature and our data.

### Atypical Localization is Likely Caused by a General Spatial Processing Impairment

The reduction from a spatial continuum to bi- or trimodal localization perception is the only presumably stroke-related result we observed. Three of the four patients with such a response pattern had a right-hemispheric lesion. This finding is in line with literature that associates binaural hearing deficits primarily with lesions in the right hemisphere (Dietz et al., 2014; Haeske-Dewick et al., 1996; Sonoda et al., 2001), albeit not exclusively (Tanaka et al., 1999). Specific lesion sites of these patients include cerebellum, thalamus, as well as basal ganglia. These are not auditory-specific regions but right-hemispheric lesions have been shown to be relevant for cross-modal spatial transformations (Heilman & Valenstein, 1979; Vallar & Perani, 1986).

Moreover, brain hemispheres have distinct specializations for spatial processing in general. While the right hemisphere facilitates metric or coordinate-based relations, the left hemisphere only provides categorical spatial relations (Jager & Postma, 2003; Kosslyn et al., 1989). Left hemispatial neglect is commonly interpreted as a breakdown of such right-hemispheric coordinate spatial representations, often affecting visual, auditory, and tactile space alike (e.g., Dietz et al., 2014; Gokhale et al., 2013; Ting et al., 2011). It is also clear that spatial hearing relies on a sophisticated interplay of visual and auditory space (e.g., King, 2009; Tonelli et al., 2015). Nevertheless, to our knowledge, hemisphere specific spatial mapping has not yet been related to sound localization, but it perfectly fits previously reported data and our finding that particularly right-hemispheric lesions result in bi- or trimodal categorical response patterns, even in the absence of overt clinical neglect.

### Localization is Primarily Compromised at Low Frequencies

Neither impaired cross-modal, nor impaired coordinate-based spatial processing readily explains why atypical localization occurs for low-frequency sounds but rarely for high-frequency sounds. All of the five affected listeners (stroke patients 2, 3, 8, 9, and control subject 1) have compromised localization with low-pass noise but only S9 has an atypical localization for high-frequency noise. As S9 has audiometric thresholds of 65 dB HL at 4 kHz, and even higher thresholds above 4 kHz, the high-frequency noise was presented near hearing threshold for this patient and the uniquely bimodal pattern should not be overinterpreted.

We can only speculate that the impaired low-frequency sound localization of these five listeners is caused by a different role that ITD and ILD cues have in sound localization. In normal-hearing listeners, low-frequency ITD cues are presumably integrated into higher-order, supramodal spatial reference frames to support a robust, accurate, and coherent spatial perception. On the other hand, high-frequency localization is based on ILDs that are not statically mapped to a direction but can be rapidly recalibrated following altered acoustic input, suggesting a greater capacity for compensation or recovery after brain lesions. Different binaural cues may also put a different emphasis on egocentric or allocentric reference frames, which, in turn, may be differently compromised.

Broadband noise, which provides the richest set of cues, is hard to localize for four of the five affected listeners. Likely, the impaired processing of low-frequency fine structure ITDs, which are of course present in broadband noise, was detrimental to the localization performance of these participants. It is known that, at least in normal hearing, localization of broadband sounds is dominated by low-frequency ITDs (Wightman & Kistler, 1992). Apparently, this low-frequency dominance is not even broken in this cohort, where poor low-frequency sound localization has a detrimental effect.

### Low-Frequency Localization and ITD-Based Lateralization are Sometimes Inconsistent

In three patients clear differences were found in results between the localization of low-frequency noise presented over loudspeakers and lateralization of a similar low-frequency noise presented over headphones with zero ILD and seven ITDs within the physiological range. S10 and S13 have a large right-side bias in the lateralization experiment. The bias did not re-surface in the localization data. Conversely, S9 has normal lateralization but the most extreme bimodal localization response. A fourth patient (S2) also has small differences that can be interpreted as a more subtle categorization difference and/or as a small bias difference. These inconsistencies cannot be understood within the large class of auditory processing models and theories that map ITDs and ILDs in a similar fashion either to a lateralization scale or to an azimuthal localization (e.g., Dietz et al., 2011; Faller & Merimaa, 2004; Hartmann, 2021). Within the normal hearing literature, it is acknowledged that there are likely certain differences that are not captured by the models and theories and that the detailed relation between the two is understudied (Hartmann, 2021). On the other hand, the differences we observed may not come as a total surprise to the seasoned expert of impaired binaural hearing, because, as Gallun (2021) summarizes the clinical and hearing-impaired literature, “the specific abilities of a given patient cannot be known without performing multiple behavioral and/or neurophysiological measurements of binaural sensitivity.” We identified three potential explanations for the differences:

First, the acoustic differences between the two tasks. In the headphone experiment we presented a 1-octave wide band of white noise ranging from 333 to 666 Hz with zero ILD and seven ITDs within the physiological range. Slightly differently, in case of loudspeaker presentation, we used a 2-octave wide band of pink noise ranging from 125 to 500 Hz. Of course, the ILD now changes naturally together with the ITD, but at these frequencies the ILDs are very small and, in addition, perceptually down-weighted (Wightman & Kistler, 1992). Specifically, the perceptual difference is most pronounced in the comparison of the zero ITD condition with the 0° azimuth condition, where ITD and ILD are zero in both modes. Irrespective of virtually identical interaural cues, spectral cues that may support localization only exist in case of loudspeaker presentation. For the low-frequency noises we used, these cues are perceptually irrelevant, and, if anything, they should improve localization and not make it worse. Overall, it seems very unlikely that the small differences in spectral composition between the two experiments causes the substantial response pattern differences for central presentations in some subjects.

Secondly, for S9 there is a large difference between the intracranial space in case of headphone listening and the auditory spatial representation in case of loudspeaker presentation. The encoded directional cues are dynamically mapped to positions in very different spaces. Moreover, not only the sound objects but the whole auditory space is created by the brain. This space depends on the stimulus properties (e.g., spectral cues or reflections), situational knowledge (i.e., wearing headphones or seeing loudspeakers), and it may even be shaped by the response interface and response options. We expect that the auditory spaces differ substantially in the two different tasks, mostly because of the situational knowledge. Then, different response patterns can arise either from individual differences of the created spaces, or from different mapping strategies the brain chooses to use in each space. This aspect is in line with the unique absence of a cortical map of auditory space and the highly dynamic task-dependent decoding of spatial cues (Middlebrooks, 2021). The above-suggested impaired cross-modal or coordinate-based spatial processing may cause an atypical perception in one but not in the other case.

The third aspect is concerned with the much smaller localization bias compared to the sound lateralization bias of S10 and S13. While the lateralization data was recorded in the acute, subacute, and chronic phase, the localization measurements were performed only in the chronic phase of a mild stroke, on average 10 months after stroke onset. While lateralization recovery was modest (Dietze et al., 2024), the possibility remains that neural recovery and new learning to localize with the modified circuits specifically ameliorated sound localization deficits that may have been more severe in the acute phase. However, for S13 the chronic lateralization bias may well be caused by the patients asymmetric hearing loss. The absence of a localization bias despite a lateralization bias (S10 and S13) and despite an asymmetric hearing loss (S13) is in line with the literature that describes the mammalian sound localization network as dynamic and highly adaptive, especially with respect to cortical decoding of binaural cues (Mendonça, 2014; Middlebrooks, 2021; Trapeau & Schönwiesner, 2015).

## Summary and Conclusions

We examined azimuthal sound localization in chronic-phase survivors of mild stroke who do not use hearing aids and compared their performance to that of young and age-matched control listeners. Consistent with previous literature, the majority of listeners—including most stroke patients—localized sounds with small RMSEs, indicative of a continuous mapping of auditory space. Four of the 14 stroke survivors exhibited a qualitatively different localization behavior, characterized by bi- or trimodal (left-center-right) categorical response patterns.

The atypical response patterns were observed predominantly in patients with right-hemispheric lesions, supporting earlier findings that implicate the right hemisphere in spatial auditory processing. Notably, the lesion sites associated with these deficits extended beyond classical auditory regions and included cerebellar, thalamic, and basal ganglia structures. This distribution suggests that the observed impairments reflect a disruption of general spatial processing mechanisms rather than a modality-specific auditory deficit. It is consistent with nonauditory findings of right-hemispheric specialization for coordinate-based spatial representations and that the left hemisphere only provides categorical spatial relations.

Atypical localization was largely restricted to low-frequency and broadband stimuli. For high-frequency sounds it was only observed in one patient with substantial high-frequency hearing loss. This frequency dependence supports the notion that low-frequency ITD-based localization relies on higher-order spatial reference frames that may be particularly vulnerable to stroke-related damage, whereas high-frequency ILD-based localization appears more flexible and amenable to compensation or recalibration. The dominance of low-frequency ITD cues likely explains why broadband stimuli were also difficult to localize for affected listeners.

The incongruent perception of three patients between a frontal loudspeaker and diotic headphone presentation (the latter reported in Dietze et al., 2024), underscores that spatial cue decoding is very task-dependent. Broad and involved testing is necessary to relate the responses either to peripheral hearing loss, to lesions within the auditory pathway, or to a general spatial processing deficit.

One of the five control subjects had similarly compromised left-center-right responses, which may or may not be the result of a silent stroke or another neuropathology. It showcases the need for large-scale comprehensive multimodal testing of elderly people, including brain scans and nonauditory tests of cognitive and neural health. Without such normative data quantitative or definitive conclusions are not possible with the typical time and access constraints of studies like the present.
